# Impact of vitamin D deficiency in relation to the clinical outcomes of hospitalized COVID-19 patients

**DOI:** 10.12688/f1000research.132214.2

**Published:** 2023-06-13

**Authors:** Andhika Rachman, Rizky Rahmaniyah, Andi Khomeini, Anggraini Iriani

**Affiliations:** 1 Division of Hematology and Oncology, Department of Internal Medicine, Dr. Cipto Mangunkusumo National Referral Hospital, Faculty of Medicine, Universitas Indonesia, Centra Jakarta, DKI Jakarta, 10430, Indonesia; 2 Department of Internal Medicine, Faculty of Medicine, Universitas Indonesia, Central Jakarta, DKI Jakarta, 10430, Indonesia; 3 Department of Internal Medicine, Wisma Atlet COVID-19 Emergency Hospital, North Jakarta, DKI Jakarta, 14360, Indonesia; 4 Department of Clinical Pathology, Yarsi University, Central Jakarta, DKI Jakarta, 10510, Indonesia

**Keywords:** Vitamin D, 25(OH)D, clinical outcome, COVID-19

## Abstract

**Background:** Vitamin D deficiency is an emerging public health problem that affects more than one billion people worldwide. Vitamin D has been shown to be effective in preventing and reducing the severity of viral respiratory diseases, including influenza. However, the role of vitamin D in COVID-19 infection remains controversial. This study aimed to analyze the impact of vitamin D deficiency on the clinical outcome of hospitalized COVID-19 patients.

**Methods:** A prospective cohort study was conducted among hospitalized COVID-19 patients at two COVID-19 referral hospitals in Indonesia from October 2021 until February 2022.

**Results:** The median serum 25(OH)D level in 191 hospitalized COVID-19 patients was 13.6 [IQR=10.98] ng/mL. The serum 25(OH)D levels were significantly lower among COVID-19 patients with vitamin D deficiency who had cardiovascular disease (p-value=0.04), the use of a ventilator (p-value=0.004), more severe COVID-19 cases (p-value=0.047), and mortality (p-value=0.002). Furthermore, serum 25(OH)D levels were significantly different between patients with mild and severe COVID-19 cases (p-value=0.019). Serum 25(OH)D levels in moderate and severe COVID-19 cases were significantly different (p-value=0.031). Lower serum 25(OH)D levels were significantly associated with an increased number of comorbidities (p-value=0.03), the severity of COVID-19 (p-value=0.002), and the use of mechanical ventilation (p-value=0.032). Mortality was found in 7.3% of patients with deficient vitamin D levels. However, patients with either sufficient or insufficient vitamin D levels did not develop mortality.

**Conclusions:** COVID-19 patients with vitamin D deficiency were significantly associated with having cardiovascular disease, mortality, more severe COVID-19 cases, and the used of mechanical ventilation. Lower serum 25(OH)D levels were associated with an increased number of comorbidities, COVID-19 severity, and the use of mechanical-ventilation. Thus, we suggest hospitalized COVID-19 patients to reach a sufficient vitamin D status to improve the clinical outcome of the disease.

## Introduction

Coronavirus Disease-2019 (COVID-19) is a rapidly spreading pandemic disease caused by Severe Acute Respiratory Syndrome Corona-Virus-2 (SARS-CoV-2), the seventh coronavirus that infect humans. This highly contagious virus spreads through phonation and breathing droplets or through direct contact with an infected person. ^1^^–^^3^ The disease can exhibit a wide range of symptoms, from asymptomatic to dramatic, such as hypoxia and multiorgan failure. ^1^^–^^5^ There is a lack of evidence-based data about the risk factors for the infection, as well as the most effective treatments. Current hospital-based management is focused on the excessive inflammatory response and respiratory support due to the fact that targeted antiviral therapies have not been widely accessible. ^6^

Vitamin D is a versatile steroid hormone that plays multiple roles in the body, including the regulation of bone and calcium metabolism. ^7^^,^^8^ It also supports the innate and adaptive immune systems against respiratory viruses. ^7^^,^^9^ It controls the innate immune system by stimulating the synthesis of antimicrobial peptides such as IL-37, cathelicidins, and defensivins. ^1^^,^^10^^,^^11^ Vitamin D also modulates adaptive immunity by regulating the formation of inflammatory T helper type 17 (Th17) cells toward the anti-inflammatory regulatory T cells and altering the primary pro-inflammatory cytokines, such as interferon- γ , TNF- α and IL-6. ^1^^,^^7^^,^^10^^–^^12^ This regulation is considered to be less effective in cases of vitamin D deficiency, although it might be obtained if vitamin D had reached a sufficient level. ^1^

Deficient vitamin D is a global health crisis, affecting over a billion people. ^7^^,^^13^^–^^17^ Vitamin D deficiency was widespread across Southeast Asian countries, despite extensive exposure to sunlight. ^18^ Based on current evidence, vitamin D helps prevent and mitigate the severity of viral respiratory diseases, such as influenza. ^4^^,^^7^^,^^19^^,^^20^ However, the role of vitamin D in COVID-19 infection remains unclear. ^4^^,^^7^

Understanding the clinical course of COVID-19 is crucial until a viable vaccination becomes widely accessible, due to the lack of specific therapies and the tremendous health and economic impact of the pandemic. ^1^^,^^21^ In this situation, deficient vitamin D is a modifiable risk factor due to its safety and affordability. ^1^^,^^22^^,^^23^ Therefore, the aim of this prospective cohort study was to assess the impact of vitamin D deficiency on the clinical outcome of hospitalized COVID-19 patients.

## Methods

### Study design

This study was a prospective cohort study conducted at two COVID-19 referral hospitals in Jakarta, Indonesia (National Emergency Hospital Wisma Atlet Kemayoran and Dr. Cipto Mangunkusumo General Hospital), from October 2021 until February 2022. The included subjects were COVID-19 positive (confirmed by reverse transcription-polymerase chain reaction [RT-PCR]) and admitted to the hospital; aged 18 years and older. The exclusion criteria were COVID-19 patients with clinically asymptomatic and severely affected COVID-19 patients who arrived using mechanical ventilation prior to admission. This study specifically involved subjects registered with mild, moderate, or severe disease according to WHO interim guidance at admission. The STROBE guidelines (Strengthening the Reporting of Observational Studies in Epidemiology) were followed for this study.

### Data collection

The SARS-CoV-2 infection was confirmed through positive RT-PCR obtained from nasal and oropharyngeal swabs collected. ^24^ The examination was carried out in the Biosafety Level 3-facility (BSL-3) with Biological Safety Cabinet Class II (BSC-II).

During the admission, each patient had 3–5 mL of blood collected in an acid citrate dextrose tube from a cuffed venous sample. The samples were transported to the laboratory in a cold chain for the measurement of vitamin D. Vitamin D status was evaluated by measuring serum 25(OH) D or 25-hydroxyvitamin D levels. The results were gathered using Roche Diagnostics’ Cobas e411, a competitive electrochemiluminescent protein binding assay.

According to Endocrine Society Clinical Practice Guideline, a serum 25(OH) D level of less than 20 ng/mL (50 nmol/L) was considered as deficient. ^25^ In this study, we divided serum 25(OH) D level into three categories, subjects with serum 25(OH) D levels ≤ 20 ng/mL (≤50 nmol/L) were considered as deficient, serum 25(OH) D levels 21-29 ng/mL (51-74 nmol/L) were considered as insufficient, and serum 25(OH) D levels ≥ 30 ng/mL ( ≥ 75 nmol/L) were considered as sufficient.

### Statistical analysis

Statistical Package for the Social Sciences (SPSS) version 27 for Macintosh was used to analyze the data that was collected. The serum 25(OH) D levels between two subgroups were analyzed with either Mann-Whitney U test for 2 subgroups and Kruskal-Wallis test for more than 2 subgroups.

### Ethical approval

Ethical approval for this study was granted by Ethics Committee of the Faculty of Medicine, Universitas Indonesia (ethical approval number: KET533/UN2.F1/ETIK/PPM.00.01/2021) and by the Ethics Committee of Wisma Atlet Hospital Jakarta (029/KERSDCWA/2021). The Declaration of Helsinki was implemented during this study.

## Results

This prospective cohort studies included 191 subjects. Before being enrolled, each participant signed a written consent form. The characteristics of the included subjects can be observed in Table 1. From the 191 subjects, 54.5% were female.

**Table 1.** Subject characteristics.

Variables	N = 191
Age, median [IQR]	42 [28]
Serum 25(OH) D level, median [IQR], in ng/mL	13.6 [10.98]
Sex, N (%)	
Female	104 (54.5)
Male	87 (45.5)
Body mass index (BMI), median [IQR]	22.66 [4.13]
COVID-19 categories, n (%)	
Mild	93 (48.7)
Moderate	67 (35.1)
Severe – critical	31 (16.2)
Number of comorbid, N (%)	
None	72 (37.7)
1	40 (20.9)
2	79 (41.4)
Type of comorbidities	
Type 2 DM, N (%)	63 (32.9)
Hypertension, N (%)	60 (31.5)
Cardiovascular disease, N (%)	17 (8.9)
Chronic liver disease, N (%)	6 (3.2)
Chronic kidney failure, N (%)	19 (9.9)
Cerebrovascular disease, N (%)	12 (6.3)
Malignancy, N (%)	19 (9.9)
HIV, N (%)	1 (0.6)
Autoimmune diseases, N (%)	10 (5.2)
COPD, N (%)	2 (1.1)
Vaccination status, n (%)	
Unvaccinated	60 (31.5)
One dose	2 (1)
Two doses	128 (67.1)
Three doses	1 (0.5)
Simple oxygenation, n (%)	61 (31.9)
ISARIC-4C Score, N (%)	
1 (Low risk)	109 (57)
2 (Intermediate risk)	39 (20.4)
3 (High risk)	31 (16.2)
4 (Very high risk)	12 (6.3)

Abbreviations: COPD, chronic obstructive pulmonary disease; Type 2 DM, Type 2 Diabetes Mellitus.

Subjects who had a history of diabetes mellitus, peripheral vascular disease, stroke or transient ischaemic index, cardiovascular disease, chronic obstructive pulmonary disease, chronic liver disease, and chronic kidney disease were considered to have comorbidities according to the Charlson Comorbidity Index (CCI). ^26^

The Coronavirus Clinical Characterization Consortium Mortality Score (ISARIC-4C Mortality Score) was established by the ISARIC-4C consortium as a predictor score for mortality among hospitalized COVID-19 patients. The determinant factors include sex, age, respiratory rate (RR), peripheral oxygen saturation, Glasgow Coma Scale (GCS), urea serum level, and C-reactive protein (CRP) level. In the ISARIC-4C Score, patients are classified into low-risk (0-3), intermediate-risk (4-8), high-risk (9-14), and very high-risk (15) categories. ^6^^,^^27^

Table 2 provided the significance levels of serum 25(OH) D level across all included subgroups using the chi-square (χ ^2^) analysis. Vitamin D deficiency was found in 74.4% of COVID-19 patients, including 65.4% of patients under the age of 60 and 11% of patients over the age of 60. Lower serum 25(OH) D levels were associated with an increased number of comorbidities, the severity of COVID-19, and the use of mechanical ventilation. Among 191 patients, mortality was found in 7.3% of patients with deficient vitamin D levels. However, subjects with either sufficient or insufficient vitamin D levels did not develop mortality.

**Table 2.** The categorized levels of serum 25(OH) D levels based on the influencing factors.

Variables	Categories		Serum 25(OH) D Level			*p*-value
		Total	Deficient ( ≤ 20 ng/mL)	Insufficient (21-29 ng/mL)	Sufficient (≥30 ng/mL)	
Age						0.928
	≤60 years old	164 (85.9%)	125 (65.4%)	30 (15.7%)	9 (4.7%)	
	>60 years old	27 (14.1%)	21 (11.0%)	5 (2.6%)	1 (0.5%)	
ISARIC-4C score						0.135
	1 (Low risk)	109 (57.07%)	77 (40.3%)	23 (12%)	9 (4.7%)	
	2 (Intermediate risk)	39 (20.42%)	30 (15.7%)	8 (4.2%)	1 (0.5%)	
	3 (High risk)	31 (16.23%)	27 (14.1%)	4 (2.1%)	0 (0.0%)	
	4 (Very high risk)	12 (6.28%)	12 (6.3%)	0 (0.0%)	0 (0.0%)	
Number of comorbidities						**0.03**
	0	72 (37.70%)	42 (24.1%)	17 (8.9%)	9 (4.7%)	
	1	40 (20.90%)	33 (17.3%)	6 (3.1%)	1 (0.5%)	
	2	79 (41.40%)	67 (35.1%)	12 (6.3%)	0 (0.0%)	
Type of comorbidities						
DM type II	No	128 (67.0%)	93 (48.7%)	25 (13.1%)	10 (5.2%)	0.051
	Yes	63 (33.0%)	53 (27.7%)	10 (5.2%)	0 (0.0%)	
Hypertension	No	131 (68.6%)	96 (50.3%)	25 (13.1%)	10 (5.2%)	0.072
	Yes	60 (31.4%)	50 (26.2%)	10 (5.2%)	0 (0.0%)	
Cardiovascular disease	No	174 (91.1%)	133 (69.6%)	31 (16.2%)	10 (5.2%)	0.534
	Yes	17 (8.9%)	13 (6.8%)	4 (2.1%)	0 (0.0%)	
Chronic liver disease	No	185 (96.9%)	140 (73.3%)	35 (18.3%)	10 (5.2%)	0.385
	Yes	6 (3.1%)	6 (3.1%)	0 (0.0%)	0 (0.0%)	
Chronic kidney disease	No	172 (90.1%)	128 (67.0%)	34 (17.8%)	10 (5.2%)	0.136
	Yes	19 (9.9%)	18 (9.4%)	1 (0.5%)	0 (0.0%)	
Malignancy	No	172 (90.1%)	128 (67.0%)	34 (17.8%)	10 (5.2%)	0.136
	Yes	19 (9.9%)	18 (9.4%)	1 (0.5%)	0 (0.0%)	
COPD	No	189 (99.0%)	145 (75.9%)	34 (17.8%)	10 (5.2%)	0.497
	Yes	2 (1.0%)	1 (0.5%)	1 (0.5%)	0 (0.0 %)	
HIV	No	190 (99.5%)	145 (75.9%)	35 (18.3%)	10 (5.2%)	0.856
	Yes	1 (0.5%)	1 (0.5%)	0 (0.0%)	0 (0.0%)	
Autoimmune diseases	No	181 (94.8%)	139 (72.8%)	32 (16.8%)	10 (5.2%)	0.498
	Yes	10 (5.2%)	7 (3.7%)	3 (1.6%)	0 (0.0%)	
BMI						0.435
	Underweight	103 (53.93%)	84 (44%)	13 (6.8%)	6 (3.1%)	
	Normoweight	8 (4.19%)	6 (3.1%)	2 (1.0%)	0 (0.0%)	
	Overweight	34 (17.80%)	25 (13.1%)	8 (4.1%)	1 (0.5%)	
	Obesity grade I	46 (24.08%)	31 (16.2%)	12 (6.3%)	3 (1.6%)	
Mortality						0.097
	No	177 (92.7%)	132 (69.1%)	35 (18.3%)	10 (5.2%)	
	Yes	14 (7.3%)	14 (7.3%)	0 (0.0%)	0 (0.0%)	
COVID-19 severity						**0.002**
	Mild	93 (48.69%)	62 (32.5%)	21 (11.0%)	10 (5.2%)	
	Moderate	67 (35.07%)	55 (28.8%)	12 (6.3%)	0 (0%)	
	Severe	31 (16.24%)	29 (15.2%)	2 (1.0%)	0 (0%)	
Vaccine doses						0.339
	0	60 (31.4%)	52 (27.7%)	7 (3.7%)	1 (0.5%)	
	1	2 (1.0%)	2 (1.0%)	0 (0.0%)	0 (0.0%)	
	2	128 (67.0%)	91 (47.6%)	28 (14.7%)	9 (4.7%)	
	3	1 (0.5%)	1 (0.5%)	0 (0.0%)	0 (0.0%)	
HFNC or ventilator use						**0.032**
	No	171 (89.53%)	126 (66%)	35 (18.3%)	10 (5.2%)	
	Yes	20 (10.50%)	20 (10.5%)	0 (0.0%)	0 (0%)	
**Total**		**191 (100%)**	**146 (76.4%)**	**35 (18.4%)**	**10 (5.2)**	

Abbreviations: BMI, body mass index; HFNC, High-flow nasal canule; DM type II, diabetes mellitus type II; HIV, human immunodeficiency virus; COPD, chronic obstructive pulmonary disease

Bold value denotes statistical significance.

Table 3 showed that serum 25(OH) D levels were significantly lower among COVID-19 patients with vitamin D deficiency who had cardiovascular disease, the use of a ventilator, more severe COVID-19 cases, and mortality. Mortality was found in 9.59% of COVID-19 patients with vitamin D deficiency.

**Table 3.** The effect of vitamin D deficiency among each subgroup.

Variables	Categories	N (%)	Serum 25(OH) D Level	
			Mean ± SD or median [IQR], in ng/mL	*p*-value
Age groups	≤60 years old	125 (85.62%)	11.94 [7.69]	0.186 ^a^
	>60 years old	21 (14.38%)	9.74 ± 5.15	
ISARIC-4C score	1 (Low risk)	77 (52.74%)	12.07 ± 4.55	0.067 ^b^
	2 (Intermediate risk)	30 (20.55%)	10.75 ± 4.73	
	3 (High risk)	27 (18.49%)	9.22 ± 5.09	
	4 (Very high risk)	12 (8.22%)	10.97 ± 6.15	
Number of comorbidities	0	46 (31.51%)	12.30 [8.06]	0.133 ^c^
	1	33 (22.60%)	10.90 ± 4.60	
	2	67 (45.89%)	10.52 ± 5.12	
Type of comorbidities				
DM type II	No	93 (63.7%)	11.05 ± 4.79	0.675 ^d^
	Yes	53 (36.30%)	11.41 ± 5.12	
Hypertension	No	96 (65.75%)	11.21 ± 4.84	0.914 ^d^
	Yes	50 (34.25%)	11.12 ± 5.05	
Cardiovascular disease	No	133 (91.1%)	11.44 ± 4.85	**0.040** ^**d**^
	Yes	13 (8.90%)	8.52 ± 4.72	
Chronic kidney disease	No	128 (87.7%)	11.49 ± 4.72	0.051 ^a^
	Yes	18 (12.3%)	7.75 [9.56]	
Malignancy	No	128 (87.67%)	11.37 ± 4.93	0.211 ^d^
	Yes	18 (12.33%)	9.83 ± 4.56	
BMI	Underweight	84 (57.53%)	11.73 [8.09]	0.082 ^c^
	Normoweight	6 (4.11%)	7.97 ± 3.54	
	Overweight	25 (17.12%)	12.47 ± 4.90	
	Obesity grade I	31 (21.23%)	9.93 ± 4.94	
Mortality rate	No	132 (90.41%)	11.58 ± 4.80	**0.002** ^**d**^
	Yes	14 (9.59%)	7.44 ± 4.26	
COVID-19 severity	Mild	62 (42.47%)	11.78 ± 4.62	**0.047** ^**c**^
	Moderate	55 (37.67%)	12.04 [6.64]	
	Severe	29 (19.86%)	7.37 [8.84]	
HFNC or ventilator use	No	126 (86.30%)	11.66 ± 4.70	**0.004** ^**a**^
	Yes	20 (13.70%)	6.39 [7.99]	

Abbreviations: BMI, body mass index; DM type II, diabetes mellitus type II; HFNC, High-flow nasal canule.

Bold value denotes statistical significance.

^a^ Analyzed using Mann-Whitney U test.

^b^ Analyzed using ANOVA test.

^c^ Analyzed using Kruskal-Wallis test.

^d^ Analyzed using t-test.

**Figure 1.**
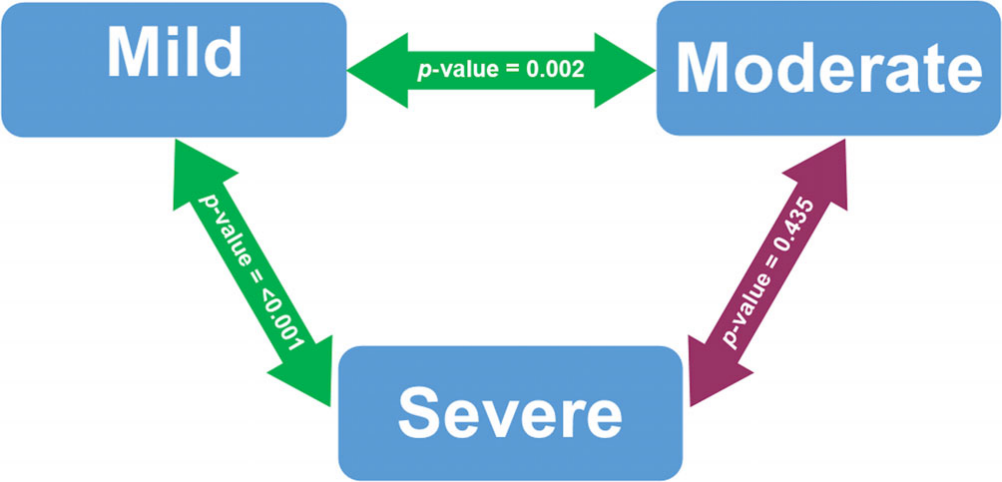
The bivariate analysis of serum 25(OH) D levels based on COVID-19 severity among deficient vitamin D subjects after adjusted with Bonferroni correction. Analyzed using Mann-Whitney U test. Significant at p-value < 0.05. The green double-arrow denotes statistically significant difference. The red double-arrow denotes non-statistically significant difference.

Figure 1 presented the bivariate analysis performed on COVID-19 patients who were vitamin D deficiency. Serum 25(OH) D levels were significantly different between patients with mild and severe COVID-19 cases (p-value < 0.001). Serum 25(OH) D levels in mild and moderate COVID-19 cases were also significantly different (p-value 0.002).

## Discussion

Prior studies indicates that Indonesia has a high prevalence of deficient vitamin D (60%), despite being located in a tropical zone where sunlight is abundant all year around. ^28^^–^^30^ Whereas the skin’s absorption of sunlight is established as the primary source of vitamin D, other variables, including age, comorbidities, and skin pigmentation, may alter the vitamin D level. ^15^^,^^30^ Based on the skin’s sensitivity to ultraviolet (UV) light, the majority of Indonesians have either Fitzpatrick skin phototype IV (with medium to dark brown) or phototype V (dark brown). A lower vitamin D is associated with darker skin pigmentation due to the higher melanin present in darker skin. ^18^^,^^30^^,^^31^ Other factors, including haze, altitude, and air pollution, also alter the ultraviolet B radiation. ^30^^,^^32^

The beneficial effect of vitamin D to reduce the severity of respiratory tract infection remains controversial. ^4^^,^^7^ The current study investigated the impact of vitamin D deficiency to the clinical outcome of hospitalized patients at two COVID-19 referral hospitals in Indonesia. We found that compared to insufficient and sufficient, those with deficient vitamin D status had more number of comorbidities ( Table 2). In COVID-19 patients with deficient vitamin D were significantly associated with cardiovascular disease ( Table 3). Our findings was supported by de la Guía-Galipienso *et al.*, that revealed vitamin D deficiency may play a critical role in the initiation of inflammation, myocardial calcification, and endothelial dysfunction, which are risk factors for cardiovascular disease. ^25^^,^^33^ The vitamin D receptor (VDR) and the enzyme 1α-hydroxylase, which are necessary for the formation of vitamin D’s active form, are expressed in cardiomyocytes, vascular endothelial cells, fibroblasts, and smooth muscle cells. ^34^^–^^37^ Left ventricular hypertrophy, vascular dysfunction, and arterial stiffness have been associated with vitamin D deficiency. A deficiency of the vitamin D receptor causes an increase in left ventricular mass and elevated levels of atrial natriuretic peptide, as well as cardiac metalloproteases and disturbances in homeostasis. Furthermore, the development of fibrotic extracellular matrix induces left ventricular dilation. ^25^^,^^38^^,^^39^

Vitamin D has been shown to have a number of beneficial effects on the cardiovascular system, including natriuretic peptide secretion, inhibition of the renin-angiotensin-aldosterone system (RAAS), anti-hypertrophic effects, and inhibition of cardiomyocyte proliferation. ^34^^,^^40^^,^^41^ Calcitriol and its analogues activate VDR, which directly suppresses angiotensin I expression and local angiotensin II synthesis in myocardial, kidney tissue, and renal arteries. ^34^^,^^42^ Studies have revealed that vitamin D enhances the anti-hypertensive effects of angiotensin 1–7 by inducing the production of angiotensin-converting enzyme 2 (ACE2). ^34^^,^^43^^,^^44^ MiR-106b-5p, which acts on juxtaglomerular cells to boost renin synthesis, has been shown to be directly influenced by VDR-deficient immune cells. ^34^^,^^44^

Moreover, vitamin D affects the progression of HF through modulating the production of metalloproteinases. Evidence strongly suggests that vitamin D has an anti-inflammatory effect by preventing nuclear factor kappa B (NF-κB) and promoting the production of IL-10, which have a significant role in the progression of CVD. ^34^^,^^45^^,^^46^ Vitamin D deficiency induces arterial stiffness and endothelial dysfunction in blood vessels, which in turn leads to enhanced inflammation, endothelial cell malfunction, and atherogenesis. ^25^^,^^47^

The severity of COVID-19 were significantly associated with the lower serum 25(OH) D levels ( Tables 2, 3 and Figure 1). Vitamin D is an immunomodulatory hormone with antibacterial and anti-inflammatory properties, and it plays a crucial role in the immune system. Vitamin D has been reported to exert its effects against COVID-19 by limiting the viral transmission, diminishing viral replication, and optimizing viral clearance. ^25^^,^^48^ Vitamin D boosts the innate immune response and protects against excessive inflammation, by increasing anti-inflammatory IL-10 and decreasing pro-inflammatory cytokines and tumor necrosis factor alpha (TNF-α). ^27^^,^^48^^–^^52^ According to the research of Daneshkhah *et al.*, a lack of vitamin D raises C-reaction protein (CRP) levels, which in turn elevates the risk of a cytokine storm. ^25^^,^^53^ The protective effects of vitamin D on the coagulation pathway led to a reduced risk of acute respiratory distress syndrome and thrombosis. ^48^^,^^54^^–^^56^ Thus, increasing vitamin D levels to adequate levels may help to prevent COVID-19 infection and complications. ^25^^,^^48^^,^^49^^,^^54^^,^^57^

Futhermore, Sabico *et al.* that conducted a multi-center randomized clinical trial in Middle East, a region with high prevalence of vitamin D deficiency, revealed that a daily oral supplementation of 5000 IU vitamin D3 for 2 weeks reduced the recovery time for gustatory sensory loss and cough among patients with mild to moderate COVID-19 symptoms and sub-optimal vitamin D status. ^58^

To the best of our knowledge, this is the first study that analyse the ISARIC-4C score in a group of patients with vitamin D deficiency. We found that serum 25(OH) D levels had no significant association with ISARIC-4C Score ( Tables 2, 3). In contrast with study by Wellbelove *et al.* that concluded the ISARIC-4C mortality score is good predictors for 30-day mortality in COVID-19 (AUROC of 0.74–0.88). ^59^ The ISARIC-4C consortium established the ISARIC-4C Mortality Score to predict the mortality of hospitalized COVID-19 patients. Multicentre cohort study was conducted among 74,944 participants at 260 different hospitals. However, the ISARIC-4C has been internally validated but not externally validated. Hence, further study is warranted to fully understand the potential of ISARIC-4C as a prognostic tool to classify patients into specific management groups. ^6^^,^^27^^,^^60^

Serum 25(OH) D levels were significantly lower among subjects that used the ventilator ( Tables 2, 3). Among all patients, mortality was found in 7.3% of patients with deficient vitamin D levels. However, patients with either sufficient or insufficient vitamin D levels did not develop mortality ( Table 2). Serum 25(OH) D levels in vitamin D deficiency subjects were significantly lower in the COVID-19 patients with mortality status ( Table 3). Our findings were consistent with the cohort study by Angelidi *et al.* and the single-center retrospective study by Alguwaihes *et al.*, which discovered that lower 25(OH) D levels were associated with increased mechanical ventilation needs and mortality risk among hospitalized patients. ^61^^,^^62^

Prior studies have revealed that vitamin D deficiency has been correlated to a 58% increased risk of acute respiratory infection, prolonged mechanical ventilation, and a 10-fold increase in mortality risk. ^61^^,^^63^^,^^64^

As a steroid hormone, vitamin D interacts with the vitamin D receptor located in the nucleus of cells to have physiologic effects. ^17^^,^^61^ The interaction of 25(OH) D with other steroid hormone receptors may have physiological effects similar to glucocorticoids. ^61^^,^^65^ Although the underlying mechanisms of vitamin D’s protection against severe COVID-19 are unknown, it is established that vitamin D reduces the production of proinflammatory cytokines such as Th1, TNF-α, interferon-β, IL-6, and promotes the production of anti-inflammatory responses such as T regulatory cells and Th2. ^50^^,^^61^^,^^66^^–^^68^ There are several explanations for vitamin D’s beneficial effects on critically ill patients. Initially, critically ill patients who are given vitamin D supplements will have their plasma vitamin D concentrations restored. Furthermore, vitamin D regulates the synthesis of immune system effector molecules such as β-defensin and cathelicidin, which are both antimicrobial peptide. ^50^^,^^61^^,^^69^^,^^70^ Cathelicidin enhances the production of anti-inflammatory cytokines while decreasing the synthesis of pro-inflammatory cytokines. As a result, vitamin D deficiency could increase the risk of sepsis and inflammation in severely ill patients by diminishing the immune response and modulatory effects on innate immunity. ^71^^–^^76^

The strength of this study lies in the fact that it is the first study to analyze the ISARIC-4C Score in COVID-19 patients with deficient vitamin D. The majority of this study’s data were collected during the Omicron variation’s development and can be utilized to make comparisons to the Delta variant or any other variants. However, this study has several limitations that should be considered to improve the further research. First, this study did not include a healthy control group as a reference population. Second, after patients were discharged, serum 25(OH) D levels were not measured. Third, the observational design and small sample size could potentially miss an important finding in this present study.

Hence, despite these limitations, our prospective cohort study demonstrates that hospitalized COVID-19 patients with vitamin D deficiency had a higher risk of using mechanical ventilation and mortality from respiratory failure and other complications. Additionally, a prior meta-analysis revealed that people with a deficient vitamin D level had an increased risk of SARS-CoV-2 infection and COVID-19-related hospitalization. Our data are consistent with the findings of recent pilot studies and a meta-analysis showing that a sufficient vitamin D status is able to reduce COVID-19 severity, indicating that it may be beneficial in minimizing the clinical and economic burden associated with COVID-19. ^1^^,^^77^^–^^79^

## Conclusion

We found that lower serum 25(OH) D levels were associated with an increased number of comorbidities, COVID-19 severity, and the use of mechanical ventilation. COVID-19 patients with vitamin D deficiency status were significantly associated with having cardiovascular disease, mortality, more severe COVID-19 cases, and the used of high-flow nasal canule (HFNC) or ventilator. This prospective cohort study doesn’t diminish the significance of the continuing vaccine effort against the health-economic burden of SARS-CoV-2 infection. As a result, we strongly suggest achieving sufficient vitamin D status, which may serve as an important adjuvant strategy to improve clinical outcomes before vaccines become widely available.

## Data Availability

Figshare: Impact of Vitamin D Deficiency in Relation to the Clinical Outcomes of Hospitalized COVID-19 Patients, DOI: https://doi.org/10.6084/m9.figshare.22145768.v2. ^80^ This project contains the following data:
- The data here is only for research paper validation of corresponding author Andhika Rachman entitled: “Impact of Vitamin D Deficiency in Relation to the Clinical Outcomes of Hospitalized COVID-19 Patients”. The raw data consists of subjects characteristics and the levels of serum 25-hydroxy-vitamin D of hospitalized COVID-19 patients. Data are available under the terms of the Creative Commons Zero “No rights reserved” data waiver (CC0 1.0 Public domain dedication).
