# Supplementary material for: The association between vitamin D deficiency and the clinical outcomes of hospitalized COVID-19 patients
Source: F1000Res. 2024 Feb 5;12:394. Originally published 2023 Apr 13. [Version 4] doi: 10.12688/f1000research.132214.4 (PMC10905025; doi:10.12688/f1000research.132214.4)
Supplement: Peer review history

# Reviewer report: Reviewer response for version 4

**DOI:** 10.5256/f1000research.162135.r243971

**Published:** 2024-02-27

**Authors:** Luigi di Filippo

**Affiliations:** 1 Institute of Endocrine and Metabolic Sciences, Università Vita-Salute San Raffaele, IRCCS Ospedale San Raffaele, Milan, Italy

Approved

Is the work clearly and accurately presented and does it cite the current literature?

Partly

If applicable, is the statistical analysis and its interpretation appropriate?

Yes

Are all the source data underlying the results available to ensure full reproducibility?

Yes

Is the study design appropriate and is the work technically sound?

Yes

Are the conclusions drawn adequately supported by the results?

Partly

Are sufficient details of methods and analysis provided to allow replication by others?

Yes

Reviewer Expertise:

endocrine diseases

I confirm that I have read this submission and believe that I have an appropriate level of expertise to confirm that it is of an acceptable scientific standard.

# Reviewer report: Reviewer response for version 3

**DOI:** 10.5256/f1000research.155718.r233579

**Published:** 2024-01-19

**Authors:** Guo-Xun Chen

**Affiliations:** 1 Department of Nutrition, University of Tennessee, Knoxville, USA; 2 College of Food Science and Technology at Huazhong Agricultural University, Huazhong Agricultural University, Wuhan, Hubei, China

The association studies like this does not ensure that there is a role of vitamin D (VD) deficiency in COVID-19 outcomes. VD deficiency has been associated with many diseases. However, its supplementation appears to lack conclusions. It will be helpful to show that VD supplementation will help outcomes of COVID-19. Without it, the value of this study is limited.

Is the work clearly and accurately presented and does it cite the current literature?

Yes

If applicable, is the statistical analysis and its interpretation appropriate?

I cannot comment. A qualified statistician is required.

Are all the source data underlying the results available to ensure full reproducibility?

Yes

Is the study design appropriate and is the work technically sound?

Partly

Are the conclusions drawn adequately supported by the results?

Partly

Are sufficient details of methods and analysis provided to allow replication by others?

Partly

Reviewer Expertise:

Biochemistry and nutrition.

I confirm that I have read this submission and believe that I have an appropriate level of expertise to state that I do not consider it to be of an acceptable scientific standard, for reasons outlined above.

# Reviewer report: Reviewer response for version 3

**DOI:** 10.5256/f1000research.155718.r233590

**Published:** 2024-01-04

**Authors:** Luigi di Filippo

**Affiliations:** 1 Institute of Endocrine and Metabolic Sciences, Università Vita-Salute San Raffaele, IRCCS Ospedale San Raffaele, Milan, Italy

Dear Editor,

 thanks for the opportunity to revise the manuscript “Impact of vitamin D deficiency in relation to the clinical outcomes of hospitalized COVID-19 patients” submitted by Dr. Rachman et al.

 The authors aimed to evaluate the impact of vitamin D deficiency on COVID-19 outcomes and reported lower vitamin D as potential risk factor, consistently with the most available literature.

 The authors had the novelty to use ISARIC-4C Score in assessing COVID-19 severity.

 The main limitation of the study is in assessment of vitamin D levels at admission in hospital where a proper exclusion of effects of disease in lowering vitamin D levels as an effect of reverse causality was not possible. Moreover, given the known associations and relationships between vitamin D levels and comorbidities, and, at the same time, comorbidities and COVID-19 outcomes, it is not possible to exclude that vitamin D should be represent only an epiphenomenon typically present in those with a more severe disease.

 Besides these limitations, that should be mentioned in the paper, the authors clearly show that lower vitamin D levels at admission represent a strong and reliable factor predicting worse outcomes. In this light, authors should mention that vitamin D levels, evaluated at admission in hospital, were previously demonstrated to prospectively predict worse outcomes in both those with severe and non-severe disease (therefore, excluding the reverse causality effect) also in multivariate analyses (therefore, excluding the effect of concomitant comorbidities) (DOI: 10.1007/s12020-023-03331-9).

 In addition, should be of interest for the readers, to mention the novelties regarding vitamin D role in Long COVID and COVID-19 vaccination (DOI: 10.1210/clinem/dgad207; DOI: 10.1210/clinem/dgad327 ; DOI: 10.1007/s12020-023-03481-w).

 Thanks.

Is the work clearly and accurately presented and does it cite the current literature?

Partly

If applicable, is the statistical analysis and its interpretation appropriate?

Yes

Are all the source data underlying the results available to ensure full reproducibility?

Yes

Is the study design appropriate and is the work technically sound?

Yes

Are the conclusions drawn adequately supported by the results?

Partly

Are sufficient details of methods and analysis provided to allow replication by others?

Yes

Reviewer Expertise:

endocrine diseases

I confirm that I have read this submission and believe that I have an appropriate level of expertise to confirm that it is of an acceptable scientific standard, however I have significant reservations, as outlined above.

## References

[1] : Lack of vitamin D predicts impaired long-term immune response to COVID-19 vaccination. *Endocrine* .2023;82(3) : 10.1007/s12020-023-03481-w 536-541 10.1007/s12020-023-03481-w 37592162 PMC10618322

[2] : Low vitamin D levels predict outcomes of COVID-19 in patients with both severe and non-severe disease at hospitalization. *Endocrine* .2023;80(3) : 10.1007/s12020-023-03331-9 669-683 10.1007/s12020-023-03331-9 36854858 PMC9974397

# Reviewer report: Reviewer response for version 3

**DOI:** 10.5256/f1000research.155718.r205417

**Published:** 2023-10-17

**Authors:** Parvaiz Koul

**Affiliations:** 1 Department of Pulmonary Medicine, Sher-i-Kashmir Institute of Medical Sciences, Srinagar, Jammu and Kashmir, India

While the authors have made changes and updated the references, the moot question of hospitalising mild cases of COVID remains unanswered and that in my opinion is a significant enough reason for me to recommend 'Not Approved' for the revision too.

Is the work clearly and accurately presented and does it cite the current literature?

Partly

If applicable, is the statistical analysis and its interpretation appropriate?

Yes

Are all the source data underlying the results available to ensure full reproducibility?

No

Is the study design appropriate and is the work technically sound?

Partly

Are the conclusions drawn adequately supported by the results?

Partly

Are sufficient details of methods and analysis provided to allow replication by others?

No

Reviewer Expertise:

NA

I confirm that I have read this submission and believe that I have an appropriate level of expertise to state that I do not consider it to be of an acceptable scientific standard, for reasons outlined above.

# Reviewer report: Reviewer response for version 2

**DOI:** 10.5256/f1000research.148441.r197568

**Published:** 2023-08-29

**Authors:** Parvaiz Koul

**Affiliations:** 1 Department of Pulmonary Medicine, Sher-i-Kashmir Institute of Medical Sciences, Srinagar, Jammu and Kashmir, India

The manuscript provides an analysis of vitamin D levels in 189 patients with COVID-19 in Indonesia and correlates the vitamin D levels with the outcomes of illness using ISARIC-4C mortality score among others. While the concept of the study is good in a tropical country with high sunlight exposure, there are some issues in design that make me uncomfortable for the interpretation of the results.

1. The study has been put forth as a 'cohort' study. However it obviously is not a longitudinal one and hence needs to be classified as an observational cross sectional study.
2. The biggest issue in design is as to why were patients with mild COVID 19 illness included and hospitalized. No guidelines recommend hospital admission of mild cases of COVID 19 and inclusion of these cases involves a serious issue in design which would clearly affect the outcomes of the patients and the results of the study. Inclusion and the very hospitalization of such cases is as such flawed and seriously affects the results. 
3. The details of the ISARIC-$C Mortality score should normally be a part of the Methods section. While the authors mention this as the first usage in COVID-19 studies, they have not unfortunately described this in the appropriate section of the manuscript.
4. An important study from India is missing from the 'Discussion' section. The study is also from an area where we expect abundance of sunlight too. In this study (Dhar A, Mir H, Koul PA. Vitamin D Levels and Length of Hospitalization in Indian Patients With COVID-19: A Single-Center Prospective Study. Cureus. 2022 Jul 9;14(7):e26704. doi: 10.7759/cureus.26704. PMID: 35959182; PMCID: PMC9359910.) of 200 patients, there was no statistically significant difference in the length of hospital stay between patients with normal serum vitamin D (VDS) and those with VDD, median LOS being 12 days (95% CI: 10, 12 days) in VDD cases and 11 days (95% CI: 10,13 days) in VDS cases (p = 0.176). The authors concluded that In Indian patients, baseline vitamin D levels are not associated with the length of hospital stay, need for mechanical ventilation, or mortality.
  Such discrepant studies need to be part of the discussion of a study like the one that authors have conducted and the possible reasons for such dichotomy proposed. 

Is the work clearly and accurately presented and does it cite the current literature?

Partly

If applicable, is the statistical analysis and its interpretation appropriate?

Yes

Are all the source data underlying the results available to ensure full reproducibility?

No

Is the study design appropriate and is the work technically sound?

Partly

Are the conclusions drawn adequately supported by the results?

Partly

Are sufficient details of methods and analysis provided to allow replication by others?

No

Reviewer Expertise:

Pulmonary Medicine

I confirm that I have read this submission and believe that I have an appropriate level of expertise to state that I do not consider it to be of an acceptable scientific standard, for reasons outlined above.

## References

[1] : Vitamin D Levels and Length of Hospitalization in Indian Patients With COVID-19: A Single-Center Prospective Study. *Cureus* .2022;14(7) : 10.7759/cureus.26704 e26704 10.7759/cureus.26704 35959182 PMC9359910

# Response

**Published:** 2023-09-06

**Authors:** Andhika Rachman

**Affiliations:** Internal Medicine, Universitas Indonesia, Jakarta, DKI Jaya, Indonesia

Dear Prof. Parvaiz Koul,

 We sincerely appreciate the time and consideration you have provided in reviewing our manuscript.

 We would like to confirm that we have made revisions to the manuscript using the “track changes” system.

 If you have any further suggestion, please don’t hesitate to contact us.

 Sincerely,

 Andhika Rachman, PhD

 Medical staff, Division of Hematology and Medical Oncology, Department of Internal Medicine, Faculty of Medicine, Universitas Indonesia - Dr. Cipto Mangunkusumo General Hospital

 The manuscript provides an analysis of vitamin D levels in 189 patients with COVID-19 in Indonesia and correlates the vitamin D levels with the outcomes of illness using ISARIC-4C mortality score among others. While the concept of the study is good in a tropical country with high sunlight exposure, there are some issues in design that make me uncomfortable for the interpretation of the results.

**1. **The study has been put forth as a 'cohort' study. However it obviously is not a longitudinal one and hence needs to be classified as an observational cross sectional study.

**Response:**

 The reviewer did raise a very important concern which will improve our manuscript. We agree with the reviewer’s constructive comments that our study is an observational cross sectional study.

**2. **The biggest issue in design is as to why were patients with mild COVID 19 illness included and hospitalized. No guidelines recommend hospital admission of mild cases of COVID 19 and inclusion of these cases involves a serious issue in design which would clearly affect the outcomes of the patients and the results of the study. Inclusion and the very hospitalization of such cases is as such flawed and seriously affects the results.

**Response:**

 We appreciate your constructive suggestions. We have provided additional explanations regarding the inclusion criteria in both the methods and discussion sections.

 During the initial phase of the pandemic, Indonesia encountered a challenging situation as it emerged as the first and enduring epicenter. The incidence of positive cases and deaths has experienced a significant surge, leading to the highest numbers in the Southeast Asian region. The Indonesian government has established a national policy that emphasizes emergency hospitals, which are intended to isolate mild cases and stop community transmission. This Indonesian government strategy has been proven to approach disease containment successfully in Wuhan, China, which was reflected by a significantly reduced COVID-19 mortality rate. ^^Thus, this study includes patients who have been registered with mild, moderate, or severe disease in accordance with the policy established by the Indonesian government. ^82^

 Furthermore, several COVID-19 pandemic studies contained some substantial biases and were not representative of the real-world conditions of the COVID-19 pandemic. ^84,85^ In regions where authorities implemented home isolation and social distancing measures, individuals with mild to moderate cases of COVID-19 were generally not hospitalized. ^84,86^ In contrast, in urban areas experiencing a high prevalence of COVID-19 cases and facing constraint intensive care resources, particularly mechanical ventilation, the majority of the hospital admissions were primarily for the individuals with severe or critical cases of COVID-19 ^.84,87,88,89^ Hospitalized people from a single center or a few centers were unlikely to represent the distribution of COVID-19 cases in an area. Also, there were evident selection biases in why and where people were hospitalized in these studies. ^84,90,91^ Multiple studies have reported the presence of censoring among subjects who were still hospitalized leading to potential biases in the interpretation of the findings. ^84,87,92^ On the contrary, we were able to identify every COVID-19 patient in our area, all of whom were admitted to the hospital. We also conducted a comprehensive follow-up for all the patients. The characteristics of our subjects were more similar to they who were exposed to the SARS-CoV-2 infection in a non-epidemic setting. As the result, our study provided a significant potential for widespread applicability and reflected the real-world conditions of the COVID-19 pandemic. ^84,93,94,95^

**Reference:**

 82. Susanto AD, Rozaliyani A, Prasetyo B, Agustin H, Baskoro H, Arifin AR, et al. Epidemiological and Clinical Features of COVID-19 Patients at National Emergency Hospital Wisma Atlet Kemayoran, Jakarta,Indonesia. Kesmas: National Public Health Journal. 2021;16(1):11-16. 10.21109/kesmas.v0i0.5233

 83. Dhar A, Mir H, Koul PA. Vitamin D Levels and Length of Hospitalization in Indian Patients With COVID-19: A Single-Center Prospective Study. Cureus. 2022 Jul 9;14(7):e26704. 10.7759/cureus.26704

 84. Xu J, Wang W, Ye H, Pang W, Pang P, Tang M, et al. A predictive score for progression of COVID-19 in hospitalized persons: a cohort study. NPJ Prim Care Respir Med. 2021 Jun 3;31(1):33.

 85. Wynants L, Van Calster B, Collins GS, Riley RD, Heinze G, Schuit E, et al. Prediction models for diagnosis and prognosis of covid-19: systematic review and critical appraisal. BMJ. 2020. 10.1136/bmj.m1328 

 86. Gandhi RT, Lynch JB, del Rio C. Mild or Moderate Covid-19. New England Journal of Medicine. 2020 Oct 29;383(18):1757–66. 10.1056/NEJMcp2009249

 87. Galloway JB, Norton S, Barker RD, Brookes A, Carey I, Clarke BD, et al. A clinical risk score to identify patients with COVID-19 at high risk of critical care admission or death: An observational cohort study. Journal of Infection. 2020 Aug;81(2):282–8. 10.1016/j.jinf.2020.05.064

 88. Di Bella S, Cesareo R, De Cristofaro P, Palermo A, Sanson G, Roman‐Pognuz E, et al. Neck circumference as reliable predictor of mechanical ventilation support in adult inpatients with COVID‐19: A multicentric prospective evaluation. Diabetes Metab Res Rev. 2021 Jan 14;37(1). ^1^

 89. Li X, Xu S, Yu M, Wang K, Tao Y, Zhou Y, et al. Risk factors for severity and mortality in adult COVID-19 inpatients in Wuhan. Journal of Allergy and Clinical Immunology. 2020 Jul;146(1):110–8. 10.1016/j.jaci.2020.04.006

 90. Liang W hua, Guan W jie, Li C chen, Li Y min, Liang H rui, Zhao Y, et al. Clinical characteristics and outcomes of hospitalised patients with COVID-19 treated in Hubei (epicentre) and outside Hubei (non-epicentre): a nationwide analysis of China. European Respiratory Journal. 2020 Jun;55(6):2000562. 10.1183/13993003.00562-2020

 91. Bialek S, Bowen V, Chow N, Curns A, Gierke R, Hall A, et al. Geographic Differences in COVID-19 Cases, Deaths, and Incidence — United States, February 12–April 7, 2020. MMWR Morb Mortal Wkly Rep. 2020 Apr 17;69(15):465–71.

 92. Gong J, Ou J, Qiu X, Jie Y, Chen Y, Yuan L, et al. A Tool for Early Prediction of Severe Coronavirus Disease 2019 (COVID-19): A Multicenter Study Using the Risk Nomogram in Wuhan and Guangdong, China. Clinical Infectious Diseases. 2020 Jul 28;71(15):833–40. 10.1093/cid/ciaa443

 93. Verity R, Okell LC, Dorigatti I, Winskill P, Whittaker C, Imai N, et al. Estimates of the severity of coronavirus disease 2019: a model-based analysis. Lancet Infect Dis. 2020 Jun;20(6):669–77

 94. Wu JT, Leung K, Bushman M, Kishore N, Niehus R, de Salazar PM, et al. Estimating clinical severity of COVID-19 from the transmission dynamics in Wuhan, China. Nat Med. 2020 Apr 1;26(4):506–10.

 95. Bialek S, Boundy E, Bowen V, Chow N, Cohn A, Dowling N, et al. Severe Outcomes Among Patients with Coronavirus Disease 2019 (COVID-19) — United States, February 12–March 16, 2020. MMWR Morb Mortal Wkly Rep. 2020 Mar 27;69(12):343–6.

**3. **The details of the ISARIC-$C Mortality score should normally be a part of the Methods section. While the authors mention this as the first usage in COVID-19 studies, they have not unfortunately described this in the appropriate section of the manuscript.

**Response:**

 Thank you for your helpful suggestions. We have added and revised the details of the ISARIC-4C Mortality Score in the Method section of the manuscript.

 Characteristics examined in the ISARIC-4C mortality score were obtained from each included patient during their admission, as defined by Knight et al. ^81^ The determinant factors include sex, age, respiratory rate (RR), peripheral oxygen saturation (%), Glasgow Coma Scale (GCS) score, urea serum (mmol/L), and C-reactive protein (mmol/L; CRP). ^6^^ ,^^27^^ ^The total scores were categorized into low risk (score 0–3), intermediate risk (score 4–8), high risk (score 9–14), and very high risk (score ≥ 15). ^81^

**Reference:**

 6. Mohamed RAE, Abdelsalam EM, Maghraby HM, et al.: Performance Features and Mortality Prediction of the 4C Score Early in COVID-19 Infection: A Retrospective Study in Saudi Arabia. *J. Investig. Med.* 2022 Feb 5;70(2):421–427. 34836890 10.1136/jim-2021-001940 PMC8635889

 27. Gupta RK, Harrison EM, Ho A, et al.: Development and validation of the ISARIC 4C Deterioration model for adults hospitalised with COVID-19: a prospective cohort study. *Lancet Respir. Med.* 2021 Apr;9(4):349–359. 33444539 10.1016/S2213-2600(20)30559-2 PMC7832571

 81. Crocker-Buque T, Myles J, Brentnall A, Gabe R, Duffy S, Williams S, et al. Using ISARIC 4C mortality score to predict dynamic changes in mortality risk in COVID-19 patients during hospital admission. PLoS One. 2022 Oct 12;17(10):e0274158

**4. **An important study from India is missing from the 'Discussion' section. The study is also from an area where we expect abundance of sunlight too. In this study (Dhar A, Mir H, Koul PA. Vitamin D Levels and Length of Hospitalization in Indian Patients With COVID-19: A Single-Center Prospective Study. Cureus. 2022 Jul 9;14(7):e26704. doi: 10.7759/cureus.26704. PMID: 35959182; PMCID: PMC9359910.) of 200 patients, there was no statistically significant difference in the length of hospital stay between patients with normal serum vitamin D (VDS) and those with VDD, median LOS being 12 days (95% CI: 10, 12 days) in VDD cases and 11 days (95% CI: 10,13 days) in VDS cases (p = 0.176). The authors concluded that In Indian patients, baseline vitamin D levels are not associated with the length of hospital stay, need for mechanical ventilation, or mortality.

 Such discrepant studies need to be part of the discussion of a study like the one that authors have conducted and the possible reasons for such dichotomy proposed. 

**Response:**

 Thank you for your valuable suggestions. We have improved our paper by citing the prospective study in the Indian subcontinent by Dhar et al. We have also provided the explanations for the possible reasons underlying the observed dichotomy in the discussion section.

 Interestingly, a single-center prospective study conducted in the Indian subcontinent, a region with a high prevalence of deficient vitamin D, demonstrated no statistically significant difference in the median length of stay (LOS) between patients with sufficient vitamin D and deficient vitamin D (p-value=0.176). The LOS for patients with deficient vitamin D was 12 days (95% CI: 10, 12 days), and the LOS for patients with sufficient vitamin D was 11 days (95% CI: 10, 13 days). They also showed that deficient vitamin D (defined as 25(OH)D < 30 ng/mL) in patients with COVID-19 was not associated with the length of hospital stay, the need for mechanical ventilation, or the mortality rate. ^83^ These different results could be explained by different cut-offs to define deficient vitamin D. In our study, a serum 25(OH)D level of less than 20 ng/mL (50 nmol/L) was considered deficient according to the Endocrine Society Clinical Practice Guidelines. ^25^On the other hand, most hospitalized patients with COVID-19 have numerous comorbidities, and this population tends to have lower vitamin D levels. Thus, vitamin D levels in such a setting should be interpreted with caution. ^83^

**Reference:**

 25. de la Guía-Galipienso F , Martínez-Ferran M, Vallecillo N, et al.: Vitamin D and cardiovascular health. *Clin. Nutr.*2021 May;40(5):2946–2957. 33397599 10.1016/j.clnu.2020.12.025 PMC7770490

 83. Dhar A, Mir H, Koul PA. Vitamin D Levels and Length of Hospitalization in Indian Patients With COVID-19: A Single-Center Prospective Study. Cureus. 2022 Jul 9;14(7):e26704. 10.7759/cureus.26704

 If you have any suggestions, we would greatly appreciate hearing them. Thanks for your attention. We eagerly await your response.

# Reviewer report: Reviewer response for version 2

**DOI:** 10.5256/f1000research.148441.r178400

**Published:** 2023-06-14

**Authors:** Shaun Sabico

**Affiliations:** 1 Department of Biochemistry College of Science, King Saud University, Riyadh, Riyadh Province, Saudi Arabia

I commend the authors for satisfactorily addressing the comments. I have no further suggestions.

Is the work clearly and accurately presented and does it cite the current literature?

Partly

If applicable, is the statistical analysis and its interpretation appropriate?

Yes

Are all the source data underlying the results available to ensure full reproducibility?

No source data required

Is the study design appropriate and is the work technically sound?

Yes

Are the conclusions drawn adequately supported by the results?

Yes

Are sufficient details of methods and analysis provided to allow replication by others?

Partly

Reviewer Expertise:

Nutrition, medical sciences, vitamin D, metabolism

I confirm that I have read this submission and believe that I have an appropriate level of expertise to confirm that it is of an acceptable scientific standard.

# Reviewer report: Reviewer response for version 1

**DOI:** 10.5256/f1000research.145122.r169546

**Published:** 2023-05-11

**Authors:** Shaun Sabico

**Affiliations:** 1 Department of Biochemistry College of Science, King Saud University, Riyadh, Riyadh Province, Saudi Arabia

The present prospective study by Rachman and colleagues analyzed the impact of vitamin D deficiency among covid-19 patients and found that those who had more comorbidities and had more severe manifestations of covid-19 were more likely to be vitamin D deficient. Although the data itself is not new, this is one of the very few studies coming from Southeast Asia where vitamin D deficiency is not expected to be as pronounced in other regions. The use of ISARIC-4C mortality score is also an added novelty. Despite this fact, their findings largely echo previous observations. I have several comments:

 Major:

1. Please add more details how covid-19 was diagnosed with reference. Was it through nasopharyngeal swab? And where was vitamin D measured? Was it in a BSL3 facility?
2. The statistics could have been expanded to control for confounders such as age and BMI. These were not explicitly mentioned in the data analysis. Furthermore, with the number of variables measured, the p-value should have been Bonferroni adjusted unless the authors prove this wasn’t necessary.
3. Lastly, the study design appears to be cross-sectional and not prospective. The observational design and small sample size have limited the findings to at best, suggestive. The limitation section could have been expanded taking into consideration the points raised.

Minor

1. Several studies that support your findings from the Middle East where deficiency is very pronounced are suggested to be added including clinical trials on vitamin D and covid-19. Similar findings from different regions and ethnic groups reinforce the role of vitamin D in severity of covid-19.

Is the work clearly and accurately presented and does it cite the current literature?

Partly

If applicable, is the statistical analysis and its interpretation appropriate?

Yes

Are all the source data underlying the results available to ensure full reproducibility?

No source data required

Is the study design appropriate and is the work technically sound?

Yes

Are the conclusions drawn adequately supported by the results?

Yes

Are sufficient details of methods and analysis provided to allow replication by others?

Partly

Reviewer Expertise:

Nutrition, medical sciences, vitamin D, metabolism

I confirm that I have read this submission and believe that I have an appropriate level of expertise to confirm that it is of an acceptable scientific standard, however I have significant reservations, as outlined above.

## References

[1] : Effects of a 2-Week 5000 IU versus 1000 IU Vitamin D3 Supplementation on Recovery of Symptoms in Patients with Mild to Moderate Covid-19: A Randomized Clinical Trial. *Nutrients* .2021;13(7) : 10.3390/nu13072170 10.3390/nu13072170 34202578 PMC8308273

[2] : Vitamin D status of Arab Gulf residents screened for SARS-CoV-2 and its association with COVID-19 infection: a multi-centre case-control study. *J Transl Med* .2021;19(1) : 10.1186/s12967-021-02838-x 166 10.1186/s12967-021-02838-x 33902635 PMC8072076

[3] : Severe vitamin D deficiency is not related to SARS-CoV-2 infection but may increase mortality risk in hospitalized adults: a retrospective case-control study in an Arab Gulf country. *Aging Clin Exp Res* .2021;33(5) : 10.1007/s40520-021-01831-0 1415-1422 10.1007/s40520-021-01831-0 33788172 PMC8009930

# Response

**Published:** 2023-05-18

**Authors:** Andhika Rachman

**Affiliations:** Internal Medicine, Universitas Indonesia, Jakarta, DKI Jaya, Indonesia

**Response To The Reviewer:**

 Dear Dr. Shaun Sabico,

 We would like to thank you for your time and consideration in handling our manuscript.

 We want to confirm that we have revised the manuscript with the tracked changes system.

 If you have any suggestions, please don’t hesitate to contact us.

 Sincerely,

 Andhika Rachman, PhD

 Medical staff, Division of Hematology and Medical Oncology, Department of Internal Medicine, Faculty of Medicine, Universitas Indonesia - Dr. Cipto Mangunkusumo General Hospital

**Major:**

1. Please add more details how covid-19 was diagnosed with reference. Was it through nasopharyngeal swab? And where was vitamin D measured? Was it in a BSL3 facility?  

Thank you for your construction feedback. We have added more details on the methods.

 The SARS-CoV-2 infection was confirmed through positive RT-PCR obtained from nasal and oropharyngeal swabs collected. ^77^ The examination was carried out in the Biosafety Level 3-facility (BSL-3) with Biological Safety Cabinet Class II (BSC-II).

 During the admission, each patient had 3–5 mL of blood collected in an acid citrate dextrose tube from a cuffed venous sample. The samples were transported to the laboratory in a cold chain for the measurement of vitamin D.

 Reference:

 77. Torretta S, Zuccotti G, Cristofaro V, Ettori J, Solimeno L, Battilocchi L, D'Onghia A, Bonsembiante A, Pignataro L, Marchisio P, Capaccio P. Diagnosis of SARS-CoV-2 by RT-PCR Using Different Sample Sources: Review of the Literature. Ear Nose Throat J. 2021 Apr;100(2_suppl):131S-138S. doi: 10.1177/0145561320953231.

  

1. The statistics could have been expanded to control for confounders such as age and BMI. These were not explicitly mentioned in the data analysis. Furthermore, with the number of variables measured, the p-value should have been Bonferroni adjusted unless the authors prove this wasn’t necessary.

The reviewer did raise a very important concern which will improve our manuscript. We strongly agree that the reviewer’s suggestion should be considered for further researches regarding this issue. The confounders such as age and BMI could not be controlled due to the minimum sample size of the subgroups and the fact that the number of samples is unequally distributed, which has not fulfilled the criteria to conduct the analysis. Thus, we have raised the point that the small sample size has become our limitation. We plan to improve the design and participants selection in the next study.

 Furthermore, we have added Figure 1 (a bivariate analysis that has been adjusted with the Bonferoni correction). Figure 1 presents the bivariate analysis performed on COVID-19 patients with vitamin D deficiency. Serum 25(OH)D levels were significantly different between patients with mild and severe COVID-19 cases (p-value < 0.001). Serum 25 (OH) D levels in mild and moderate COVID-19 cases were also significantly different (p-value 0.002).

  

1. Lastly, the study design appears to be cross-sectional and not prospective. The observational design and small sample size have limited the findings to at best, suggestive. The limitation section could have been expanded taking into consideration the points raised.

We have added the points of observational design and small sample size as limitations of our study. Additionally, we want to verified that our study's design is cohort prospective. Even if the risk variables (vitamin D levels) are measured upon admission, the outcome is followed and observed until the patient leaves the hospital, becomes worse, requires a ventilator, or passes away. The person had not used a ventilator when first recruited.

**Minor**

1. Several studies that support your findings from the Middle East where deficiency is very pronounced are suggested to be added including clinical trials on vitamin D and covid-19. Similar findings from different regions and ethnic groups reinforce the role of vitamin D in severity of covid-19.

Thank you for your advice. We have improved our paper by citing the multi-center randomized clinical trial in the Middle East that conducted by Sabico et al. We have also cited the studies carried out by Al-Daghri et al. and Alguwaihes et al.

 Please don't be hesitant to contact us if you have any concerns or suggestions about the revised manuscript. If you have any suggestions, we would greatly appreciate hearing them. Thanks for your attention. We eagerly await your response.
